# Screening and identifying a novel M-MDSCs-related gene signature for predicting prognostic risk and immunotherapeutic responses in patients with lung adenocarcinoma

**DOI:** 10.3389/fgene.2022.989141

**Published:** 2023-01-04

**Authors:** Geng-Chong Wang, Mi Zhou, Yan Zhang, Hua-Man Cai, Seok-Theng Chiang, Qi Chen, Tian-Zhen Han, Rong-Xiu Li

**Affiliations:** ^1^ State Key Laboratory of Microbial Metabolism, School of Life Sciences and Biotechnology, Shanghai Jiao Tong University, Shanghai, China; ^2^ Department of Rheumatology, Renji Hospital, School of Medicine, Shanghai Jiaotong University, Shanghai, China

**Keywords:** LUAD, M-MDSCs, prognostic model, immunotherapeutic responses, precision medicine

## Abstract

**Background:** Lung adenocarcinoma (LUAD) shows intratumoral heterogeneity, a highly complex phenomenon that known to be a challenge during cancer therapy. Considering the key role of monocytic myeloid-derived suppressor cells (M-MDSCs) in the tumor microenvironment (TME), we aimed to build a prognostic risk model using M-MDSCs-related genes.

**Methods:** M-MDSCs-related genes were extracted from The Cancer Genome Atlas (TCGA) and Gene Expression Omnibus (GEO) databases. Utilized univariate survival analysis and random forest algorithm to screen candidate genes. A least absolute shrinkage and selection operator (LASSO) Cox regression analysis was selected to build the risk model. Patients were scored and classified into high- and low-risk groups based on the median risk scores. Gene Ontology (GO) and Kyoto Encyclopedia of Genes and Genomes (KEGG) enrichment analysis along with R packages “estimate” and “ssGSEA” were performed to reveal the mechanism of risk difference. Prognostic biomarkers and tumor mutation burden (TMB) were combined to predict the prognosis. Nomogram was carried out to predict the survival probability of patients in 1, 3, and 5 years.

**Results:** 8 genes (VPREB3, TPBG, LRFN4, CD83, GIMAP6, PRMT8, WASF1, and F12) were identified as prognostic biomarkers. The GEO validation dataset demonstrated the risk model had good generalization effect. Significantly enrichment level of cell cycle-related pathway and lower content of CD8^+^ T cells infiltration in the high-risk group when compared to low-risk group. Morever, the patients were from the intersection of high-TMB and low-risk groups showed the best prognosis. The nomogram demonstrated good consistency with practical outcomes in predicting the survival rate over 1, 3, and 5 years.

**Conclusion:** The risk model demonstrate good prognostic predictive ability. The patients from the intersection of low-risk and high-TMB groups are not only more sensitive response to but also more likely to benefit from immune-checkpoint-inhibitors (ICIs) treatment.

## Introduction

Lung cancer is the second most commonly diagnosed cancer with 11.4% of incidence rate and 18% mortality rate, ranked first, among 36 tumors in 185 countries in the worldwide (Sung et al., 2021). LUAD is a prevalent subtype of NSCLC and comprises for greater than 40% of lung cancer cases (Shi et al., 2016). ICIs is one of the most promising treatments for LUAD when compared to other cancer therapies, such as surgery, chemotherapy, and radiotherapy. Though ICIs therapy shows an increased estimated overall survival rate over 5 years among these patients which is 16% (Gettinger et al., 2018), only a small fraction of patients can response to ICIs treatment. Therefore, it is an urgent need to identify effective prognostic biomarkers to stratify the patients and predict immunotherapeutic responses for precision medicine.

Tumor heterogeneity is tightly linked to the tumor microenvironment (TME). Benefiting from the advancements in sequencing technologies and machine learning algorithms, understanding of the characteristics of TME at the molecular level has substantial clinical value to predict prognosis in patients. In the TME of LUAD, many studies have been focused on the prognosis of T cells (Du et al., 2021), B cells (Zhang et al., 2021), cancer-associated fibroblasts (CAFs) (Navab et al., 2011; Min et al., 2021), and tumor-associated macrophages (TAMs) (Chen et al., 2021), as well as the biological processes, including epithelial-mesenchymal transition (EMT) (Shi et al., 2021) and angiogenesis (Cai et al., 2021). However, the analysis on the prognostic significance of M-MDSCs, showing a strong immunosuppressive function, is still insufficient.

In clinical practice, metastasis is an important cause of cancer-related deaths (Veglia et al., 2021). MDSCs are highly undifferentiated cells derived from immature myeloid progenitor cells with immunosuppressive ability in the TME of LUAD. They can be divided into M-MDSCs and polymorphonuclear myeloid-derived suppressor cells (PMN-MDSCs) (Talmadge and Gabrilovich, 2013; Bronte et al., 2016; Gabrilovich, 2017; De Cicco et al., 2020). M-MDSCs exert greater immunosuppressive effects relative to PMN-MDSCs, which suppress antigen-non-specific and antigen-specific T cell functions by generating nitric oxide (NO), arginase-1 (Arg-1), and other immunosuppressive factors (Wang Y. et al, 2019). Moreover, M-MDSCs participate in EMT and angiogenesis in the TME, forming a pre-metastatic niche (Groth et al., 2019), and finally differentiate into TAMs with immunosuppressive ability. TAMs participate in angiogenesis and tumor pre-metastasis (Yang et al., 2020; Consonni et al., 2021). Although various methods have been developed to overcome the therapeutic resistance due to the existence of M-MDSCs, the results remain unsatisfactory.

Given the important role of M-MDSCs between monocytes and TAMs and its close relationship with tumor heterogeneity in the TME, we hypothesized that M-MDSCs-related genes could act as prognostic signature genes and effectively stratify patients. Based on univariate survival analysis, random forest algorithm, and LASSO Cox regression method, a risk model was generated using the TCGA training set and these findings were validated in the GEO dataset. We aimed to discover robust biomarkers to precisely stratify LUAD patients. Understanding the mechanism underlying the differences between risk groups might help develop effective strategies for ICIs therapy.

## Materials and methods

### LUAD and M-MDSCs datasets

The data of RNA-seq transcriptome (workflow: HTSeq-Counts) and corresponding clinical information of the TCGA-LUAD cohort (https://portal.gdc.cancer.gov/) were downloaded using the R package “TCGAbiolinks” (Colaprico et al., 2016) as the training group. Entrez IDs were converted into gene symbols and the counts were transformed using the file from TCGA (https://gdc.cancer.gov/about-data/gdc-data-processing/gdc-reference-files) into the transcripts per million (TPM) formation. Next, the data were log (x+1) normalized. Using the “GEOquery” (Davis and Meltzer, 2007), from the GEO database (https://www.ncbi.nlm.nih.gov/geo), the validation dataset GSE68465 (Director’s Challenge Consortium for the Molecular Classification of Lung Adenocarcinoma et al., 2008) and M-MDSCs datasets GSE131552 and GSE162353 (Kwak et al., 2020) were obtained. Patients with insufficient information were excluded with the exclusion criteria as follows: overall survival days less than 30 days; lack of specific information on clinical characteristics; recurrent cases; lack of information on gene expression in the clinical data. First, 594 RNA-seq cases and 515 LUAD clinical cases were extracted from the TCGA database. After the exclusion, 592 cases (including 59 normal tissues and 533 tumor tissues) for differential analysis and 482 cases for survival analysis were obtained. Both GSE131552 and GSE162353 consisted of three monocytes samples and three M-MDSC cases and the GSE68465 comprised 439 cases for survival analysis.

### Analysis of differentially expressed genes (DEGs)

DEGs were acquired between monocytes and M-MDSCs using the R package, “limma”, and visualized on a heatmap (This DEGs were defined as M-DEGs). The cut-off values for M-DEGs screening were *p* < 0.05 and |logFC| > 1. DEGs in TCGA were identified using the R package, “DESeq2”, and visualized on a volcano plot (This DEGs were defined as LUAD-DEGs). The cut-off values for LUAD-DEGs were set as padj < 0.05 and |logFC| >1. The volcano plot were drawn using the R packages, “ggplot2”. Finally, The genes obtained from the intersection of M-DEGs and LUAD-DEGs were defined as M-MDSCs-related genes, for these genes can exert function to affect prognosis and immunotherapy of LUAD patients.

### Acquisition of the signature gene

To construct an effective and precise prognostic risk model based on M-MDSCs-related genes, the “survival” package was firstly used followed by the univariate Cox regression analysis to filter the prognosis-related genes (*p* < 0.05). Subsequently, the random forest algorithm was utilized to obtain genes with the top variance using the R package, “randomForestSRC”. The intersecting genes between univariate Cox regression and random forest analysis were our target for further evaluation.

### Construction and verification of M-MDSCs-related prognostic model for patients with LUAD

The prognostic risk model was constructed by LASSO Cox regression analysis using “survival” and “glmnet” packages. Each patient was scored according to the levels of gene expression and their corresponding coefficients as follows: Risk score = Exp (gene_1_)* Coef (gene_1_) + ……+ Exp (gene_n_)* Coef (gene_n_), where Exp indicated the level of gene expression and Coef represented the corresponding coefficient of gene. According to the median risk value, patients were classified into low- and high-risk groups. To visualize the grouping effect between these two groups, t-distributed stochastic neighbor embedding (t-SNE) analysis was conducted using the “ggplot2” and “Rtsne” packages. The Kaplan-Meier (K-M) curve and log-rank test was applied to compare differences of the survival probabilities between the two risk groups using the “survival” and “survminer” packages. The receiver operating characteristic curve was plotted to evaluate the accuracy of the model using “survminer”, “timeROC”, and “survival” packages.

### Functional annotation and estimation of immune status between risk groups

To elucidate the mechanism and find potential targets between the two risk groups, GO annotation and KEGG analysis were performed using “clusterProfiler” (Yu et al., 2012). Additionally, to further estimate the immune status between the two risk groups, R packages “estimate” and “ssGSEA” were applied. These results were demonstrated using “ggplot2”.

### Evaluation of TMB for patients with LUAD

The Mutation Annotation Format (MAF) files of somatic variants for LUAD were extracted from TCGA using the GDCquery_Maf (pipelines = “varscan”) tool in the R package, “TCGAbiolinks”. The mutational data were analyzed using “maftools.” The mutational frequency with the number of variants/the length of exons (38 million) was defined as the TMB value. Further, patients were categorized into low-TMB and high-TMB groups according to the median TMB value for subsequent analysis.

### Construction and calibration of nomogram for patients with LUAD

Univariate and multivariate Cox analysis were conducted using the R package, “survival”. Four clinical variables (age, gender, risk score, and stage) were employed to construct the nomogram for predicting the overall survival of LUAD patients over 1, 3, and 5 years by using the R package, “rms.” To estimate the consistency between the practical results and prediction outcomes, a calibration curve was constructed and plotted.

### Statistical analysis

The t-test or Wilcoxon test were chosen to compare the mean between two groups based on actual requirements. Benjamini–Hochberg was carried out to adjust the *p*-value for multiple testing with the R function “p.adjust”. Kaplan-Meier (K-M) and the log-rank test were performed for the survival analysis. *p* < 0.05 represented statistical significance. All statistical analyses were performed on the R software (v4.0.3).

## Results

### Obtation of M-MDSCs-related prognostic signature genes for patients with LUAD

**FIGURE 1:**
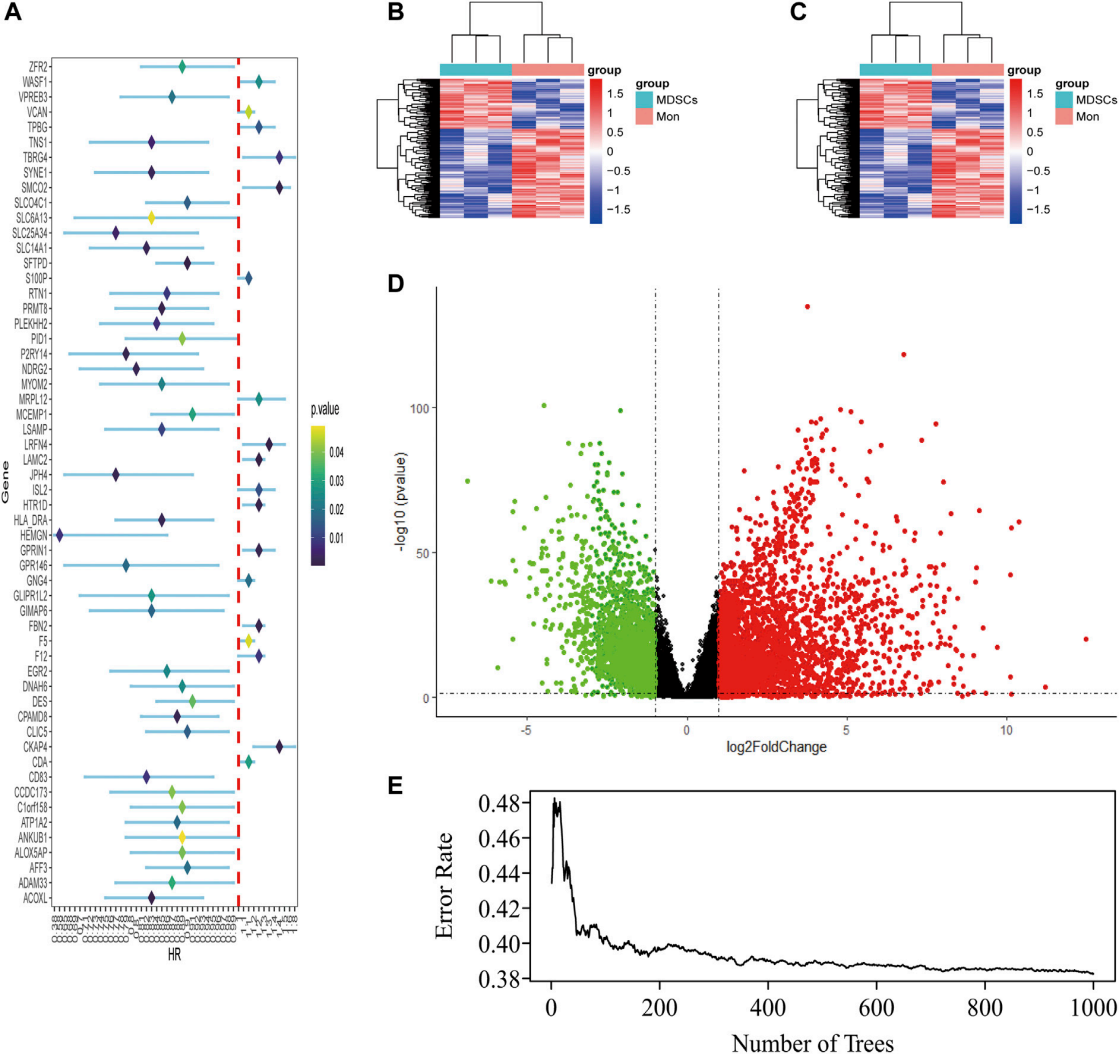
Identification of M-MDSCS-related signature genes in LUAD patients. **(A)** Univariate Cox regression analysis revealed the 56 genes significantly correlated with clinical prognois. **(B,C)** Heatmap for the difference between Monocytes and M-MDSCs datasets (GSE131552, GSE162353). **(D)** A volcano map of the differently expressed genes in TCGA training set. **(E)** Randomforest showed the number of trees and its classification effect.

**FIGURE 2:**
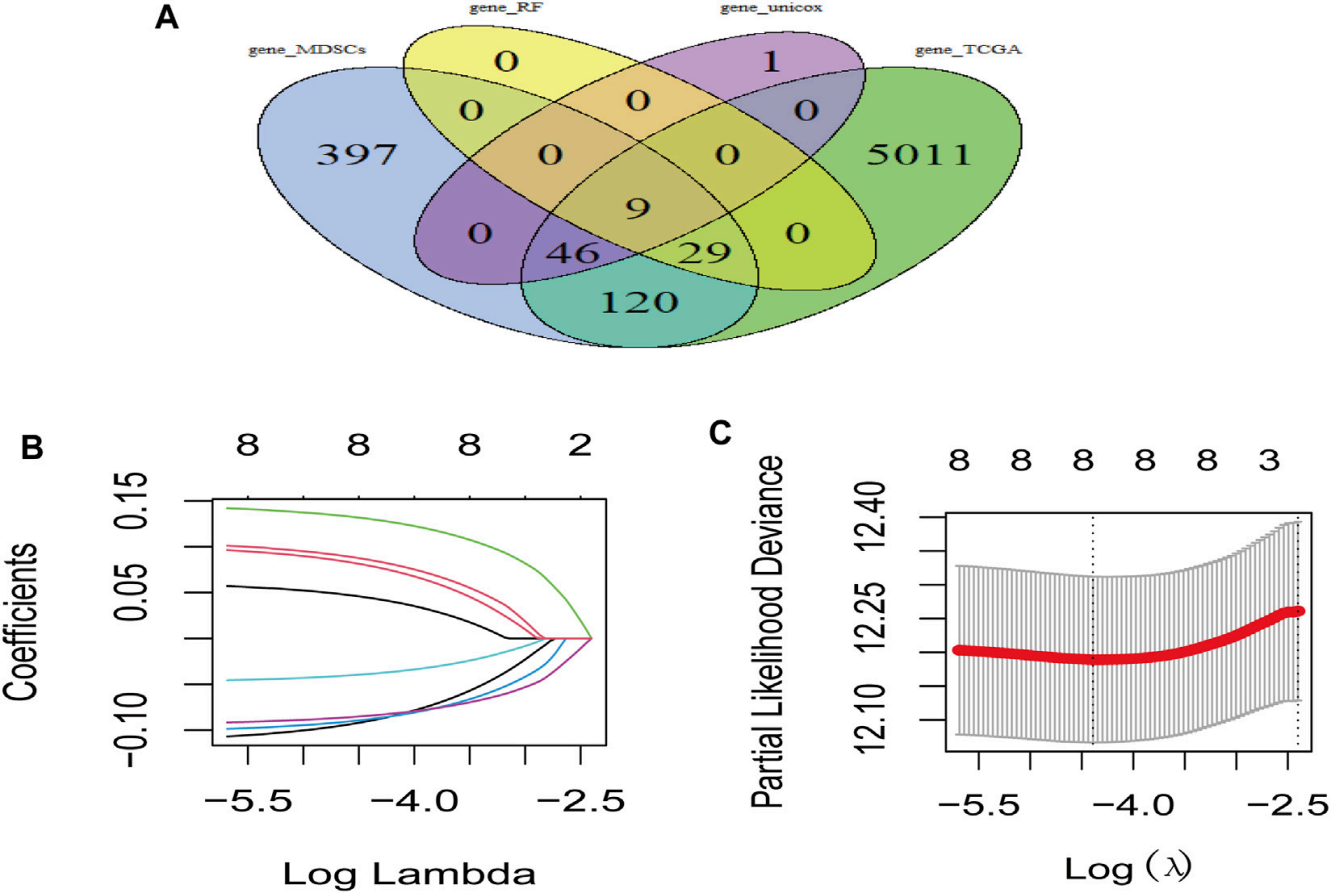
Risk model based on M-MDSCs-related signature genes for LUAD patients. **(A)** Venn plot showed genes acquired from different methods for model constrution. **(B,C)** Lasso and partial likelihood deviance coefficient profiles of the selected genes.

LUAD-DEGs between normal controls and TCGA-LUAD patients were presented (Figure 1D). M-DEGs between monocytes and M-MDSCs in the GEO dataset were shown in Figures 1B,C. In order to simplify complexities and achieve the best stratification with fewer genes, two different algorithms (including the univariate Cox regression and random forest) were utilized to select the most significant prognostic-related genes. As the result, nine genes were identified from univariate Cox regression (Figure 1A) and random forest (Figure 1E) analysis as shown in the Venn diagram (Figure 2A).

### Construction and verification of the M-MDSCs-related prognostic model for patients with LUAD

Prognostic model was constructed using the nine genes obtained from the above analysis, eight signature genes were acquired (Figures 2B,C). The risk score for each LUAD patients was derived as follows: expression values of

$$\begin{array}{c} VPREB3*\left(-0.0895214478145377\right)+LRFN4*\left(0.130109206874633\right)+F12*\left(0.0789203393477133\right)+PRMT8*\left(-0.0840653641514239\right)\\ +TPBG*\left(0.085357138019245\right)+GIMAP6*\left(-0.0384913787997157\right)+CD83*\left(-0.0878273131513041\right)+WASF1*\left(0.0439661671050762\right) \end{array}$$

**FIGURE 3:**
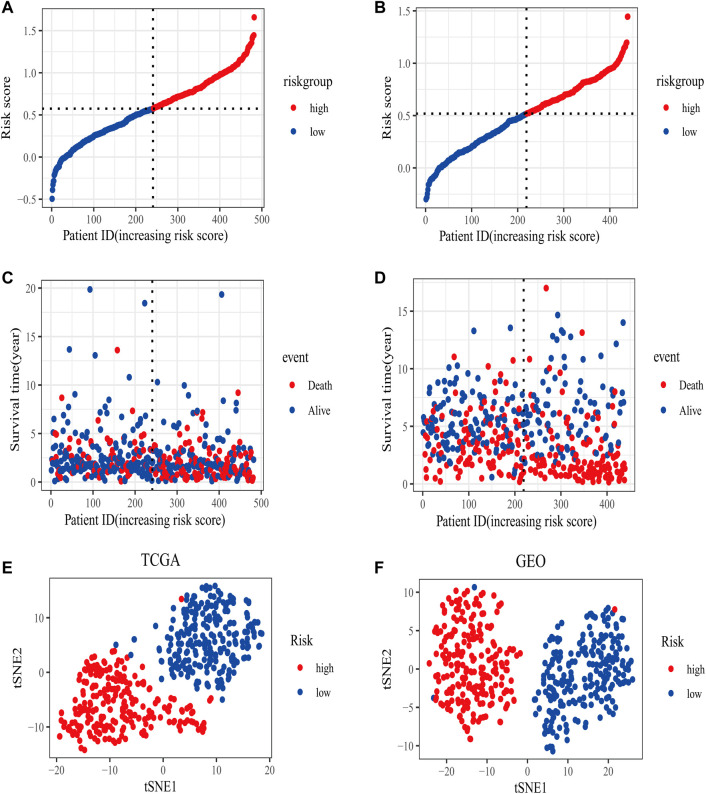
The distribution of risk scores in training (TCGA) and validation set (GSE68465). **(A)** The patients from TCGA training set were divided into high- and low-risk groups based on the median value of the risk scores. **(B)** The patients from GEO validation set were divided into high- and low-risk groups based on the median value of the risk scores. **(C)** The distribution of the survival time between high- and low-risk groups in the TCGA training set. **(D)** The distribution of survival time between high- and low-risk groups in the GEO validation set. **(E)** The t-SNE plot in the TCGA training set. **(F)** The t-SNE plot in the GEO validation set.

**FIGURE 4:**
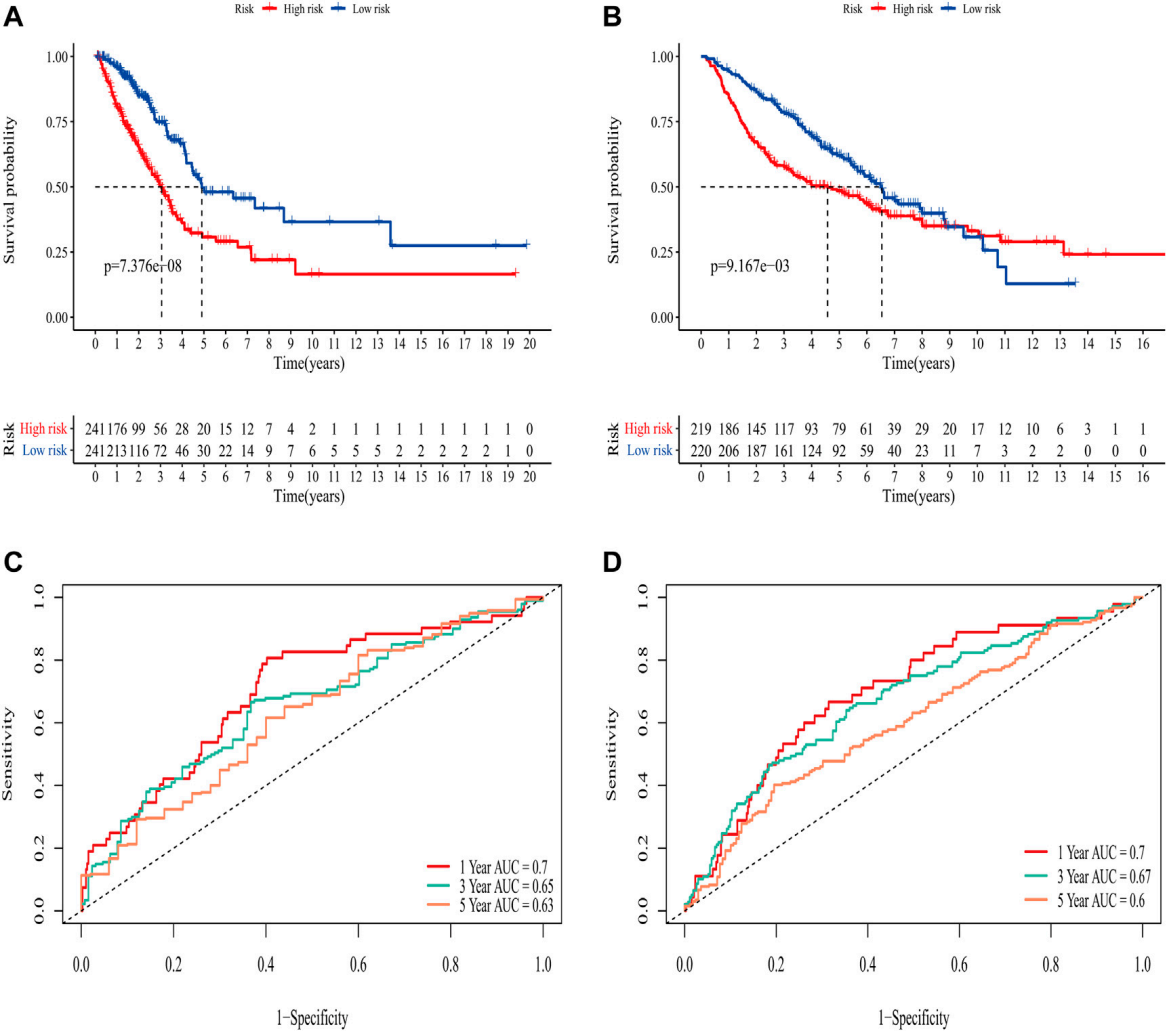
Evaluation of the predictive ability of the eight-gene signature. **(A)** K-M survival curve for OS in the TCGA training set. **(B)** K-M survival curve for OS in the GEO validation set. **(C)** Time-dependent ROC curve of prognostic model at 1-,3-,5-year in the TCGA training set. **(D)** Time-dependent ROC curve of prognostic model at 1,-3,-5- year in GEO validation set.

In the TCGA training set, LUAD patients were categorized into two risk groups (Figure 3A) based on the median value of the risk scores. Blue dots represented patients who were alive, while those in red indicated the death of patients, the survival time was obviously reduced with an increase in the risk scores (Figure 3C); The t-SNE plot demonstrated a good grouping effect between the risk groups (Figure 3E). K-M curve analysis showed significant survival differences (*p* < 0.05), whereby the low-risk group had a better prognosis (Figure 4A). The values of area under the time-dependent ROC curve over 1, 3, and 5 years were 0.7, 0.65, and 0.63, respectively (Figure 4C).

The area under the curve (AUC) of risk model in the TCGA training set was greater than 0.6, suggesting it had a good predictive power. To validate the generalization of our model, GSE68465 included 439 samples with useful survival information was applied for the following analysis. The risk scores distribution based on the median value and the association between survival time and risk scores was shown in Figures 3B,D. With an increase of the risk scores, the survival time was decreased, which was consistent with the results of the TCGA training set. t-SNE analysis showed good grouping effects between the two risk groups (Figure 3F). The K-M curve showed significant survival differences (*p* < 0.05) and patients in the high-risk group experienced worse survival outcomes (Figure 4B). The time-dependent ROC curve demonstrated good generalization effect, AUC values for the prognostic model over 1, 3, and 5 years were 0.7, 0.67, and 0.6, respectively (Figure 4D).

### Functional and pathway enrichment analyses and estimation of the immune status between two risk groups

**FIGURE 5:**
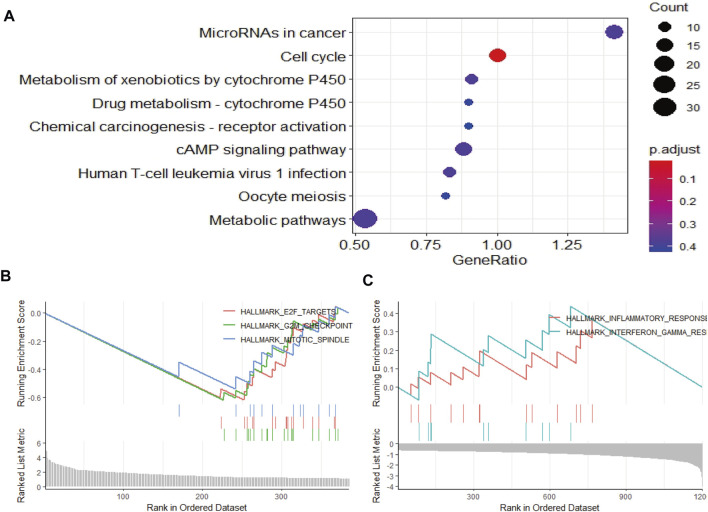
Function and pathway enrichment analysis by GSEA between high- and low-risk groups in LUAD patients. **(A–C)** The pathway enrichment and analysis between high- and low-risk groups in LUAD patients.

**FIGURE 6:**
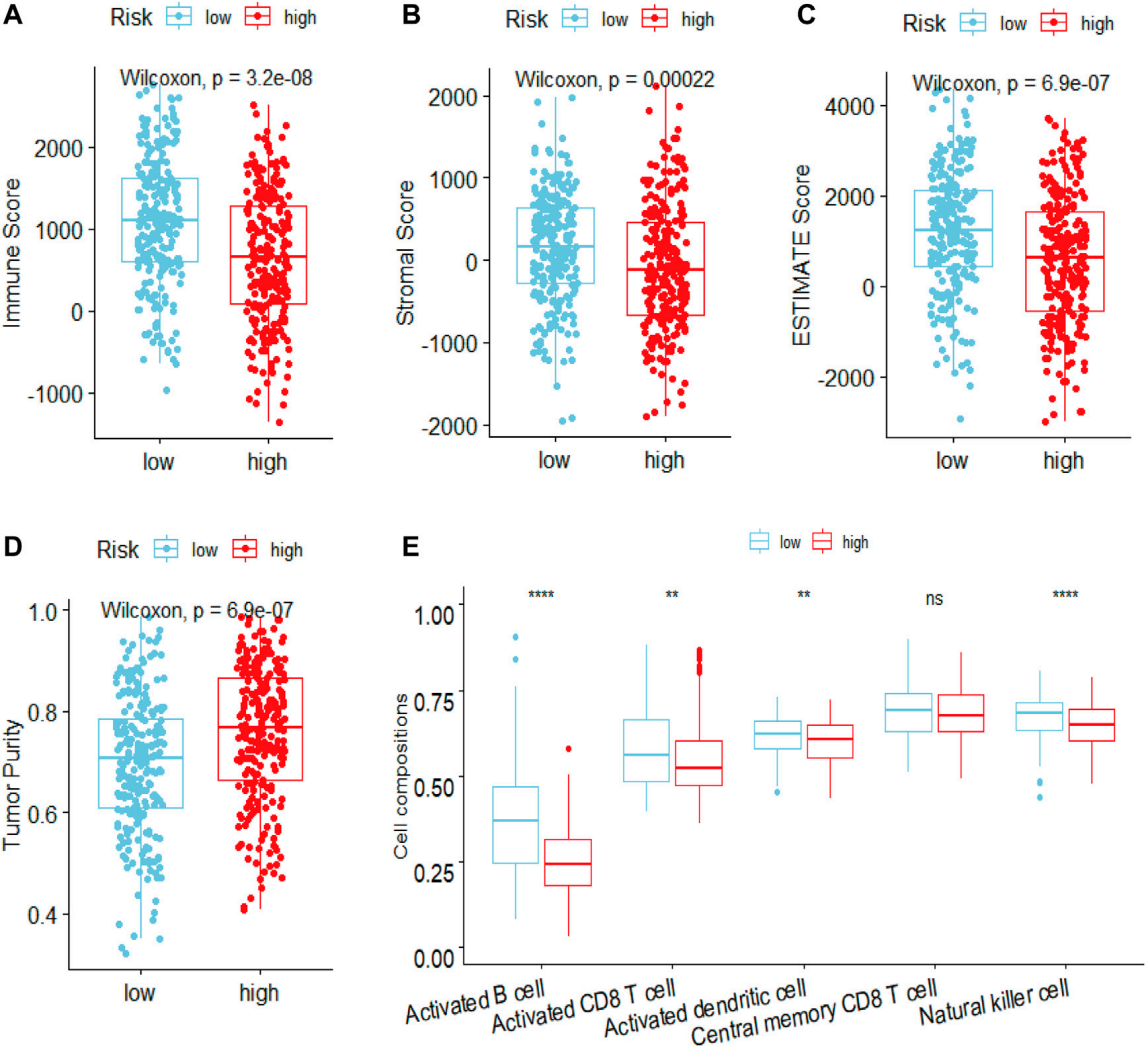
Estimated the difference of immune status between high- and low-risk groups in LUAD patients by ESTIMATE and ssGSEA algorithm. The ESTIMATE algorithm evaluated the difference of **(A)** immune scores **(B)** stromal score **(C)** estimate score **(D)** tumor purity between high- and low-risk groups in LUAD patients **(E)** ssGSEA algorithm evaluated the level of immune cells infiltration between high- and low-risk groups in LUAD patients. ***p < 0.001; **p < 0.01; *p < 0.05; ns, Not significant.

To elucidate the mechanism affecting the prognosis of LUAD patients between the two risk groups, GO annotation, KEGG enrichment analyses and immune cell infiltration status estimation were performed. The GO annotation of DEGs between the two risk groups were mainly enriched in the metabolic and multicellular organismal process (Supplementary Figure S1). KEGG results showed high-risk group significantly enriched in cell cycle processes (Figure 5A), including “E2F targets”, “G2M checkpoint” and “mitotic spindle” (Figure 5B), while low-risk group remarkably enriched in IFN-γ and inflammation related pathway (Figure 5C). Estimate algorithm was performed to compare immune status between the two risk groups (Figures 6A–D), the immune score and estimate score of low-risk group was significantly higher than high-risk group, while the tumor purity of low-risk group was remarkably lower than high-risk group. It seems the risk was consistent with the immune status. Then, ssGSEA was carried out to compare the distribution of immune cells (Figure 6E) and verified the “estimate” result. “Activated B cell”, “Activated CD8^+^T cell”, “Activated dendritic cell” and “Natural killer cell” were remarkably enriched in the low-risk group, which contributed to its decrease of risk.

### Analysis of TMB between the two risk groups

**FIGURE 7:**
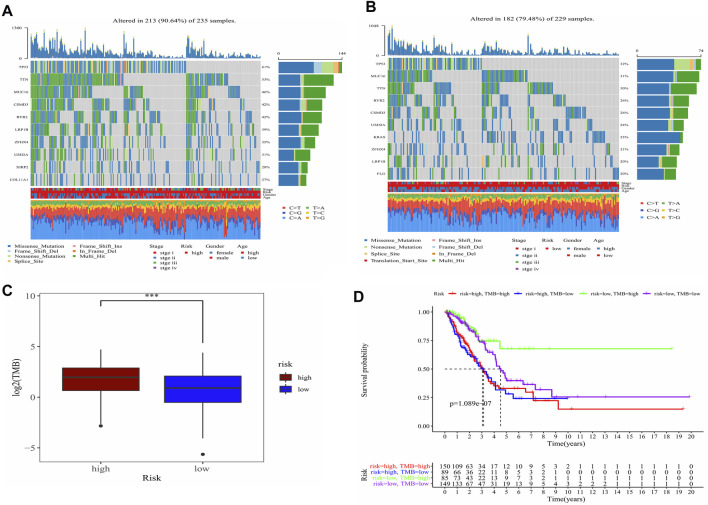
Analysis of the TMB between high- and low-risk groups and predicted prognosis in different combination in LUAD patients. **(A)** Waterfall plot demonstrated mutation information of the genes with high mutation frequencies in the high-risk group. **(B)** Waterfall plot demonstrated mutation information of the genes with high mutation frequencies in the low-risk group. **(C)** Difference of TMB between high- and low-risk groups. ***, *p* < 0.001. **(D)** K-M curve for four combinations groups divided by risk groups and TMB groups.

TMB in high-risk and low-risk groups was also investigated. The differences in the mutational landscape between the two risk groups were shown in Figures 7A,B. The frequency of mutations was higher in the high-risk group (90.64%) as compared to the low-risk group (79.48%) in the waterfall map depicting the top 10 mutations. The boxplot showed that the low-risk group had a lower TMB value relative to the high-risk group (Figure 7C) (*p* < 0.001). Analysis of overall survival indicated that the patients from the intersection between low-TMB and high-risk groups showed the worst prognosis, while patients from the intersection between high-TMB and low-risk groups showed the best prognosis (*p* < 0.0001) (Figure 7D).

### Construction of the nomogram and its calibration for patients with LUAD

**FIGURE 8:**
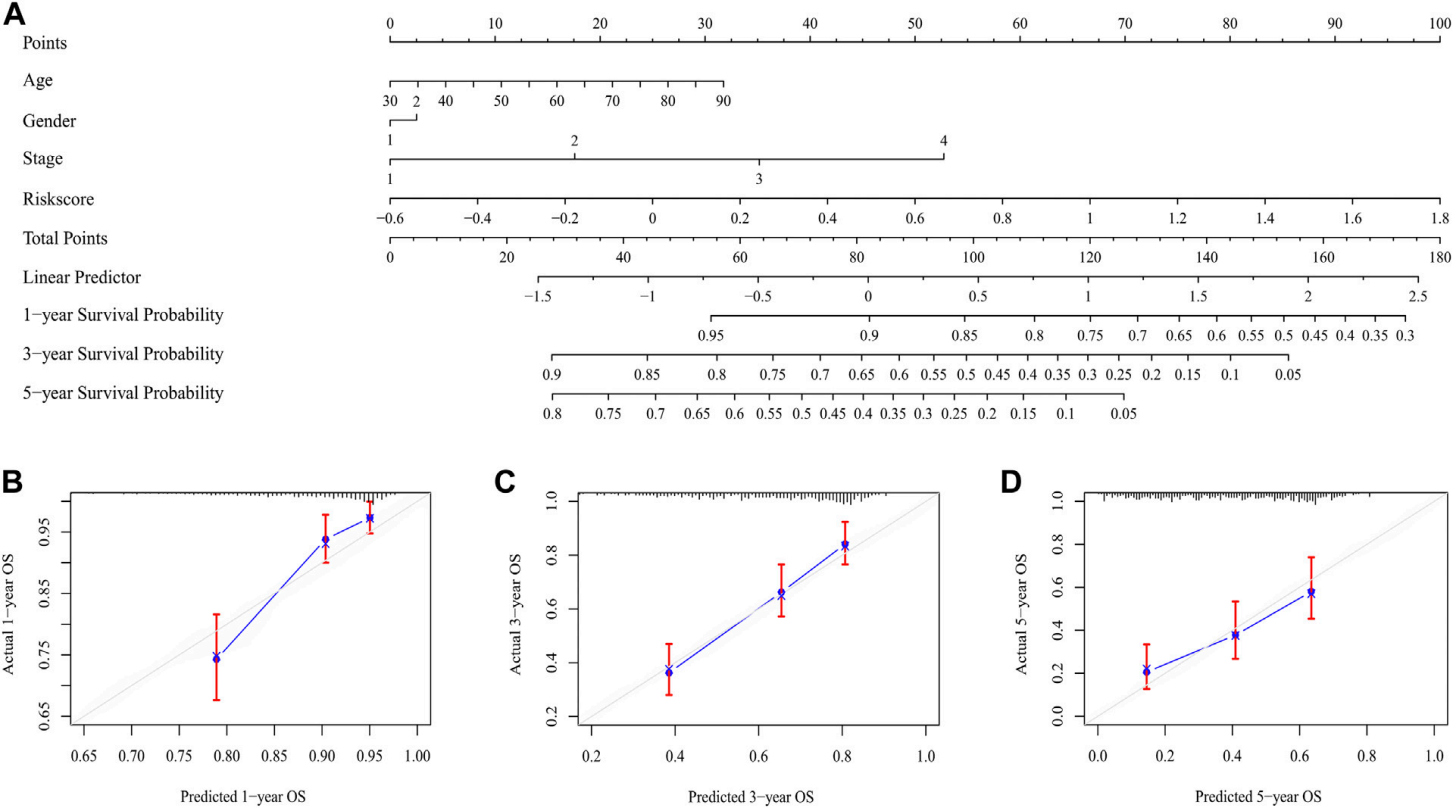
The nomogram for predicting the overall survival of LUAD patients. **(A)** The nomogram for predicting the LUAD patients with 1,-3,-5-year overall survival. **(B–D)** The plots depicted the calibration of the nomogram between predicted and actual outcomes.

The risk score was proved to be an independent prognostic factor after performed univariate and multivariate Cox regression analysis (*p* < 0.001) (Supplementary Figures S2, S3). The nomogram integrated the risk score with other clinical characteristics, including age, stage and gender for the prediction of 1-, 3-, and 5-year overall survival probabilities (Figure 8A), thus providing a quantitative tool for estimating prognosis of patients in the clinical settings. Good consistency was observed between the practical results and prediction outcomes (Figures 8B–D).

## Discussion

LUAD is a heterogeneous intratumoral disease (Herbst et al., 2018), making its high incidence and mortality rate that causes major public healthcare concern (Malla et al., 2021). Traditional clinical treatment does not consider changes at the molecular level, and a huge deficiency exists in traditional clinical treatment. Although immunotherapy has substantially improved the survival of patients with advanced LUAD, the outcome remains unsatisfactory due to the tumor heterogeneity. While the studies on the roles of heterogeneity in TME are limited, therefore, it is necessary to identify potential biomarkers of TME to stratify patients for the personalized therapy. In this study, the prognostic risk model based on M-MDSCs-related genes demonstrated good prognostic prediction ability in the TCGA training set and showed good generalization effect in the GEO validation set. High- and low-risk groups stratified by prognostic biomarkers showed significant differences in survival analysis. Functional annotations and assessment of immune cell infiltration levels revealed that the high-risk group was enriched in cell cycle-relevant targets and contained lower infiltration ratios of CD8^+^T cells, which resulted in a strong immunosuppressive state than low-risk group. The patients from the intersection between low-risk and high-TMB groups had the best prognosis. Risk score was an independent prognostic factor, the nomogram indicated that the practical results and prediction outcomes had good consistency.

In our research, prognostic biomarkers consisted of eight genes (VPREB3, TPBG, LRFN4, CD83, GIMAP6, PRMT8, WASF1, and F12), most of which were closely related to the prognosis of LUAD. VPREB3 encoded proteins were involved in the maturation of B cells and might play an important role in the assembly of pre-B cell receptors (Rosnet et al., 2004). In the TME, B cells participated in all clinical stages of lung cancer and played an important role in tumor development (Wang S.-S. et al, 2019). TPBG was a leucine-rich transmembrane glycoprotein that encoded cell adhesion, which was expressed in many tumor tissues but hardly in normal adult tissues and was involved in the directional movement of cells. TPBG, also known as 5T4, was a marker of early differentiation of human embryonic stem cells and was involved in the EMT process and was associated with poor prognosis in a variety of tumors (Stern and Harrop, 2017). LRFN4, also known as SALM3, was expressed in many tumors and leukemia cell lines. LRFN4 was involved in the migration of monocytes/macrophages to inflammatory regions and might play a role in the polarization of M2 macrophage (Konakahara et al., 2011), which were involved in a poor prognosis for LUAD (Cao et al., 2019). The CD83 gene encoded a membrane protein that belonged to the immunoglobulin superfamily of receptors, studies had shown that CD83 was not only a typical co-stimulatory molecule, but played an important role in controlling the immune response (Grosche et al., 2020). CD83 was expressed in a variety of active immune cells (B lymphocytes, T lymphocytes, monocytes, dendritic cells, neutrophils, etc.) (Grosche et al., 2020), and these immune cells were closely related to the prognosis of LUAD. GIMAP6 was expressed in lymphocytes and was involved in the development of cells in the immune system, where it regulated immune function by controlling cell death and activating T cells (Ho and Tsai, 2017). In addition, GIMAP6 induced by IFN-γ played an important role in antimicrobial immunity (Yao et al., 2022). Although GIMAP6 had been poorly reported in the prognosis of LUAD, its activation of T lymphocytes played an important role in improving the prognosis of LUAD (Jackute et al., 2015). PRMT8 was a member of the arginine methyltransferase, its participation in arginine methylation played an important role in cell signaling, RNA processing, transcriptional regulation and DNA repair (Lee et al., 2005). PRMT8 had been reported to be involved in the prognosis of a variety of tumors, with high expression of PRMT8 associated with a good prognosis in breast and ovarian cancers and poor prognosis in gastric cancer (Hernandez et al., 2017). WASF1, also known as WAVE1, was a member of the Wiskott-Aldrich syndrome protein family and acted as a regulator between Rac-GTPase and actin to induce actin polymerization (Ito et al., 2018), was an integral part of cell motility and a key step in cancer metastasis (Fernando et al., 2008), which was a hallmark of poor prognosis in patients with LUAD (Inamura and Ishikawa, 2010). F12, also known as clotting factor 12, was a serine protease. There was substantial evidence showed they played an important role in macrophage polarization and tumor-associated macrophages were associated with poor prognosis for LUAD (Renne and Stavrou, 2019; Zheng et al., 2020). We investigated and explored the role of eight signature genes in the prognosis of LUAD, which reasonably explained as prognostic biomarkers to a certain degree.

The predictive power of prognostic risk model and significant difference in survival analysis between high- and low-risk groups of LUAD patients prompted us to explore the mechanism of the risk differences. GO enrichment results were consistent with the need for high-intensity metabolic activity in tumor cells. In the TME, tumor cells rapidly proliferated in a hypoxic environment, only by producing metabolic flows different from normal cells could they meet their survival in extreme condition. KEGG enrichment showed that the high-risk group was mainly enriched in the signaling pathways related to the cell-cycle (G2M_Checkpoint, E2F_Targets, Mitotic_Spindle), while the low-risk group was mainly enriched in the signaling pathways related to IFN-γ and inflammation. The result of high-risk group was consistent with the theory that overactivated cell cycle allowed tumor cells to evade immune surveillance in addition to accelerating cell proliferation (Li and Stanger, 2020), which contributed to its high risk. IFN-γ played an important role in activating cellular immunity and activating antitumor immunity (Jorgovanovic et al., 2020), which could kill tumor cells and led to low risk. Analysis of GO and KEGG showed the risk difference was closely related to the immune status of patients. Then, we estimated the level of immune cell infiltration with R packages “estimate” and “ssGSEA.” In the TME, which includes cells that exerts immune killing effects (CD8^+^T, CD4^+^T, NK, DC, M1, etc.) and immune suppressive effects (Treg, MDSCs, TAM, etc.), there are also stromal cells (CAF, etc.) and the infiltration ratio of different cells is closely related to the prognosis of LUAD patients. In the estimation of immune cells infiltration by “estimate,” the low-risk group had a higher proportion of immune score, a higher proportion of estimation score and a lower proportion of tumor purity when compared to the high-risk group, which pointed to the close relationship between risk difference and proportion of immune cell infiltration. In order to further verify the relationship between risk difference and immune status, we evaluated the level of immune cells infiltration between high- and low-risk groups with “ssGSEA” and found that the results were consistent with the estimation by “estimate.” Compared with the high-risk group, the low-risk group had a higher proportion of immune killing-related cells, such as activated B cells, CD8^+^T cells, DC cells and NK cells. Studies have shown that activated B cells (Germain et al., 2014), CD8^+^T cells (Gueguen et al., 2021), DC cells (Goc et al., 2014) and NK cells (Zeng Y. et al, 2021) were associated with a good prognosis in LUAD patients. In addition to explaining the mechanism of the difference of risk between the high- and low-risk groups, functional annotations and immune cell infiltration levels also indicated a close relationship between the degree of risk and immune status. The high-risk group was in an immunosuppressive state due to the overactivation of the cell cycle and a lower infiltration of immune-killing cells. Besides increasing the infiltration ratio of immune-killing cells, targeting cell cycle-related target signaling pathway will achieve a better clinical effect in reversing the immunosuppressive state of high-risk group. Available data showed the prognosis of LUAD patients was significantly improved by targeting G2M_Checkpoint-related signaling pathways (Zeng L. et al, 2021). The low-risk group had relatively strong immune-killing function with a higher infiltration ratio of CD8^+^T, which contributed to a better prognosis in LUAD patients. The correlation between the invasion ratio of CD8^+^T in tumor tissues and the response to ICIs had been clinically proved (Topalian et al., 2016). Given the relationship between risk level and immune status in high- and low-risk groups, it is reasonable to infer that low-risk group is more likely to benefit from ICIs treatment.

At present, ICIs therapy utilizes the immune system to kill tumor cells and only benefit a small number of patients who can respond to this treatment (Syn et al., 2017). Inspired by this phenomenon, we try to utilize some biomarkers to stratify these patients, to overcome the shortcomings of ICIs therapy caused by tumor heterogeneity. TMB is a potential molecular predictive biomarker for ICIs response, implying that neoantigens generated by tumor cells can be effectively recognized by the immune system (McGrail et al., 2021). However, using TMB as a predictive biomarker to select patients who can respond to ICIs therapy remains unsatisfactory (Addeo et al., 2019). The risk difference stratified by the prognostic biomarkers obtained from our model may explain the imperfect forecasting of TMB. A higher TMB means that there is a greater possibility producing tumor-associated neoantigens that can be effectively recognized by the immune system and thus can be utilized to predict the effects of immunotherapy. From the distribution of TMB and immune cells infiltration between high- and low-risk groups, patients from the intersection of low-risk and high-TMB groups might produce more effective tumor-associated neoantigens, which could be identified by cytotoxic T lymphocyte and led to more CD8^+^T cells infiltration, while patients from the intersection of high-risk and low-TMB groups might not produce enough tumor-associated neoantigens, which finally resulted in lower CD8^+^T cells infiltration. Prognostic analysis of the four combinations (high-risk and high-TMB, high-risk and low-TMB, low-risk and high-TMB, low-risk and low-TMB) confirmed this deduction, the patients from the intersection of low-risk and high-TMB groups had the best prognosis and the patients from the intersection of high-risk and the low-TMB groups had the worst prognosis. Hence, in theory, patients from the intersection of low-risk and high-TMB groups are more likely to sensitive response to and benefit from ICIs therapy.

Despite the promising results, our risk model demonstrates its potential value in precision medicine, the current study still exists some shortcomings. Firstly, the prognosis of patients is closely related to the TME. Considering the heterogeneity of the TME, constructing a prognostic risk model with only one kind of signature molecules may limit the prediction ability of the prognostic risk model. Secondly, the regulation of tumor progression by these eight genes requires experimental investigation. Thirdly, the samples for the construction of the prognostic risk model gathered from retrospective studies, whether the conclusion can guide the clinic still needs a large number of multi-center clinical samples for further discussion and verification.

## Conclusion

In conclusion, the prognostic risk model constructed by M-MDSCs-related genes shows good predictive ability in the prognosis of LUAD patients. The risk stratification of patients by prognostic biomarkers demonstrates the degree of risk is closely related to immune status. Theoretically, the patients have the characteristics of both low-risk and high-TMB are not only more sensitive response to but also more likely to benefit from ICIs treatment.

## Data Availability

The datasets presented in this study can be found in online repositories. The names of the repository/repositories and accession number(s) can be found in the article/Supplementary Material.
