# Supplementary figures and images for: Screening and identifying a novel M-MDSCs-related gene signature for predicting prognostic risk and immunotherapeutic responses in patients with lung adenocarcinoma
Source: Front Genet. 2023 Jan 4;13:989141. doi: 10.3389/fgene.2022.989141 (PMC9869425; doi:10.3389/fgene.2022.989141)

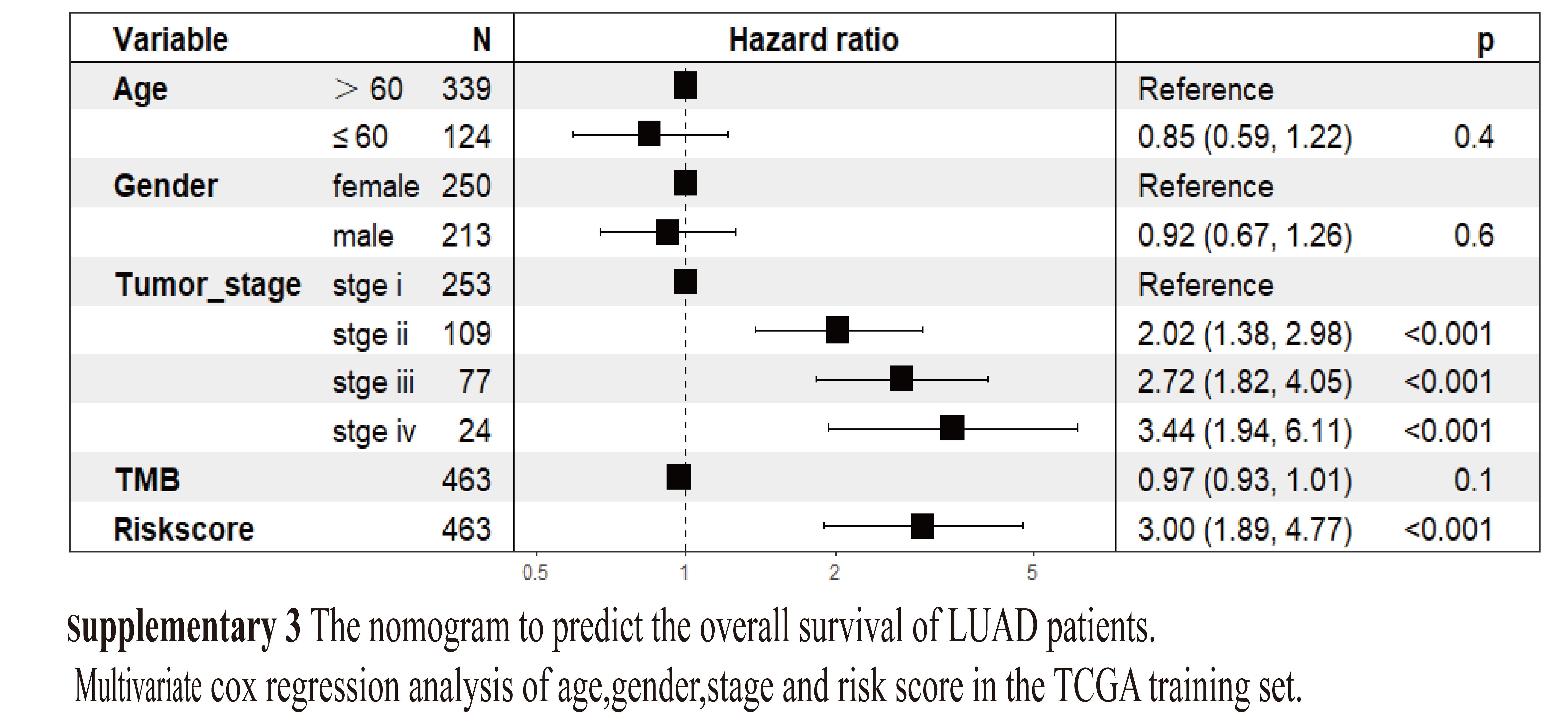

Supplement: Supplementary file 1 [file Image3.tif]

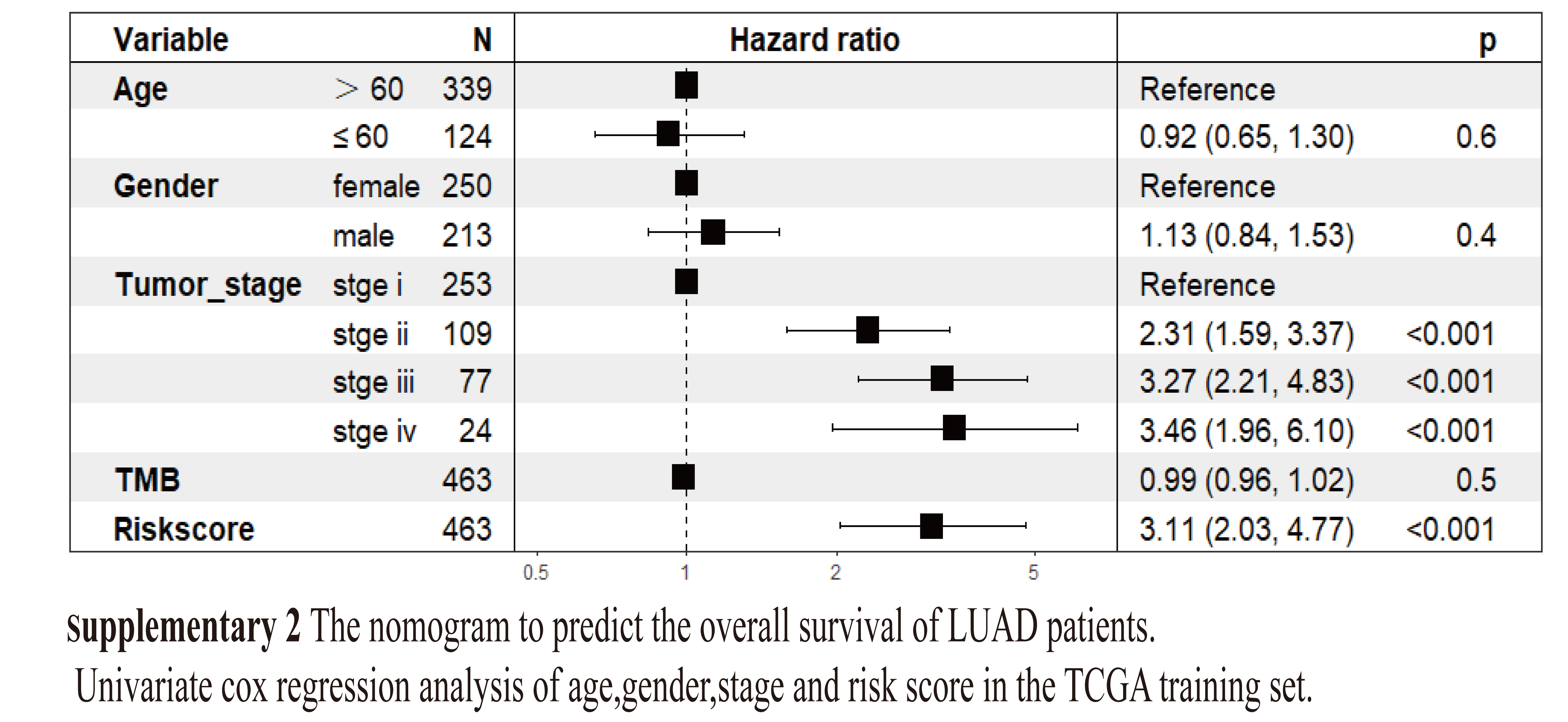

Supplement: Supplementary file 2 [file Image2.tif]

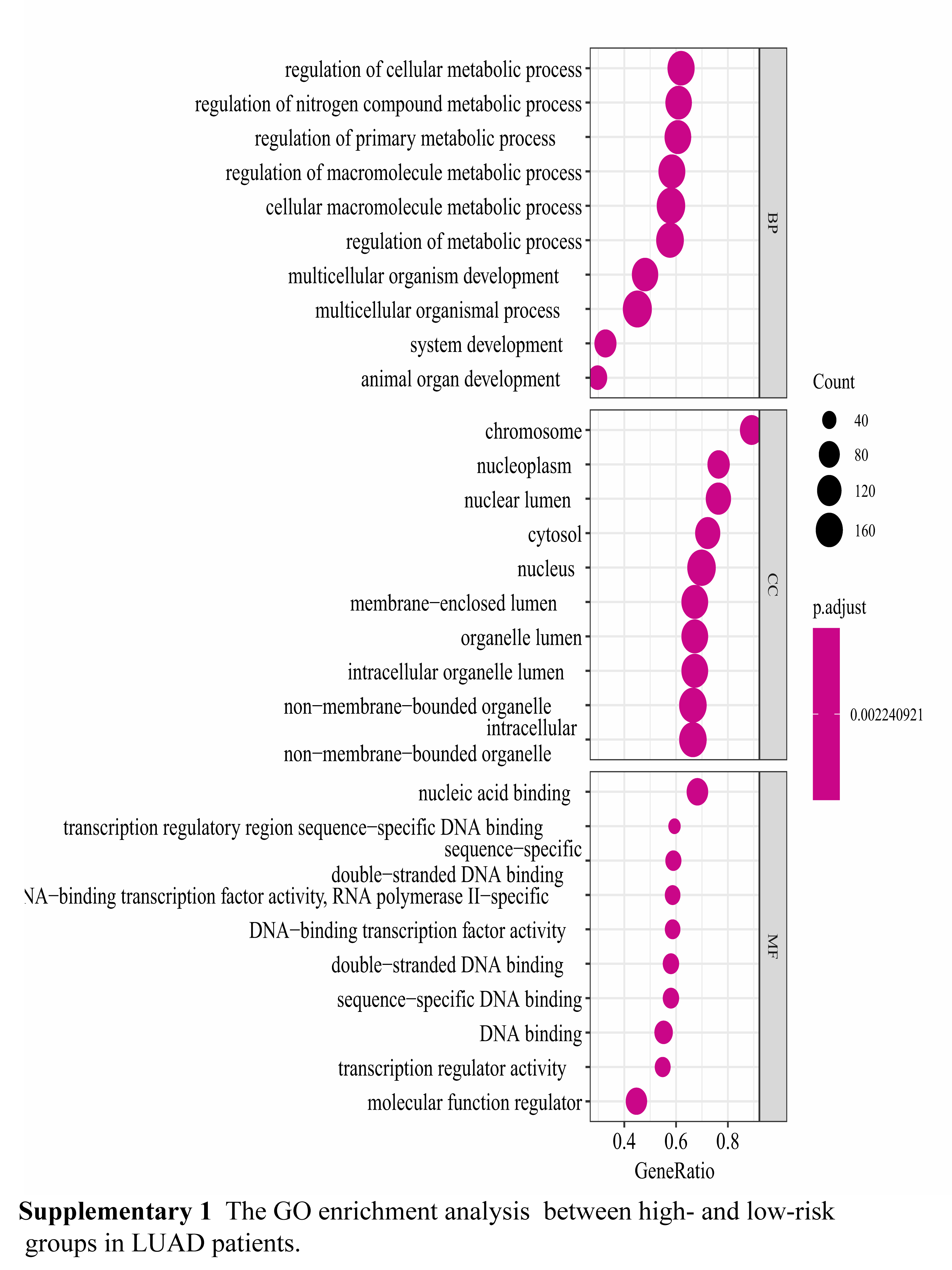

Supplement: Supplementary file 3 [file Image1.tif]
